# Piloting an Oral History Approach to Investigate Cancer Perspectives Among Residents of Appalachian Kentucky

**DOI:** 10.13023/jah.0501.07

**Published:** 2023-04-01

**Authors:** Courtney Martin, Lauren Hudson, Nathan L. Vanderford

**Affiliations:** Markey Cancer Center, University of Kentucky, courtney.martin@uky.edu; Markey Cancer Center, University of Kentucky; University of Kentucky

**Keywords:** Appalachia, cancer disparities, cancer interventions, cancer risk factors, health behaviors, oral history, social determinants of health

## Abstract

**Introduction:**

Kentucky ranks first in the U.S. in overall cancer incidence and mortality rates. Areas of the state that fall within the Appalachian Region, along Kentucky’s eastern border, experience disproportionately high rates of cancer compared to non-Appalachian counties. Purpose: This pilot study investigates whether oral history interviews can be used to understand perspectives on cancer among residents of Appalachian Kentucky.

**Methods:**

In 2020, participants (n = 5) who identified as being from and/or having strong connections to Appalachian Kentucky were recruited to participate in this pilot study. Participants included individuals working in cancer-related fields, oncology professionals, and those with personal cancer experience. Using an oral history approach, subjects were asked about challenges within Appalachia that contribute to high rates of cancer regionally. Interviews were analyzed using qualitative content analysis, and data were condensed into themes, subthemes, and subtopics. Relational content analysis was then used to illustrate relationships between the problems being faced in Appalachia and their contributing factors, with potential solutions to those problems.

**Results:**

Six key themes emerged from analysis of the oral history interviews: (1) problems being faced in Appalachia; (2) contributing factors; (3) potential solutions; (4) Appalachian disposition; (5) experiences with and thoughts on cancer; and (6) defining success v. the future without changes (intervention). A further 25 subthemes were identified from within these themes. Taken together, these themes and subthemes point to potential areas for specific intervention to shift Appalachia’s cancer burden.

**Implications:**

This pilot study demonstrates potential benefit in using oral history interviews to elucidate Appalachian Kentuckians’ perspectives on cancer. From the nuanced insights gained through this method, a set of culturally appropriate interventions were identified that could address the disproportionate cancer burden in the region. Future studies using an oral history approach could aim to reveal other specific aspects of how cancer impacts individuals, families, and communities.

## INTRODUCTION

Cancer is the second-leading cause of death in the U.S., with over 600,000 deaths projected to occur in 2023.^1^,^2^ Kentucky ranks first in the nation in cancer incidence and associated mortality.^1^,^2^ In 2023, an estimated 30,270 Kentuckians will be diagnosed with cancer and over 10,000 cancer deaths are expected to occur.^2^ Areas of the state that fall within the Appalachian Region—those counties along Kentucky’s eastern edge—experience disproportionately high rates of cancer compared to non-Appalachian counties.^3^,^4^ Residents of Appalachia are a unique, primarily Caucasian population who have a distinct culture. They are known for putting family first, valuing hard manual work, being kind-spirited, and having a deep appreciation for the place they call home.^5^,^6^

The cancer disparities in Appalachian Kentucky are driven by key social determinants of health (SDOH). SDOH are conditions/situations in which individuals live, learn, and/or work and include factors such as income and social status, education, and access to health care.^7^ These factors affect individuals’ lives in a variety of ways, including their health and overall quality of life. SDOH substantially impact Appalachians’ health and create the conditions for higher rates of cancer in the region. The cancer burden in this area is, in part, attributable to factors like the lack of access to health care, poverty, low educational attainment, distrust for those outside the region, physician shortages, lack of health literacy, and comorbidities, such as obesity and diabetes.^3^,^4^,^8^–^14^

SDOH influence high cancer rates in multiple ways. Consider lack of access to health care and lack of transportation: both factors can interplay to prevent sufficient healthcare engagement and result in delayed or inexistent treatment. With appointments being missed due to inadequate healthcare access or transport, overall health outcomes are poorer for those patients from Appalachia with a cancer diagnosis.^15^ A study investigating cancer in Appalachian Kentucky found that individuals who lived furthest from the nearest healthcare facility received more advanced-stage-cancer diagnoses than those in closer proximity.^16^ This illustrates the relationship between cancer outcomes (higher stage at diagnosis) and access to local treatment.

A pilot study was conducted to determine whether oral history interviews could be used to better understand perceptions of cancer among those residing in Appalachian Kentucky. Given Appalachian people’s traditional use of storytelling as a way to express their cultural practices and beliefs, oral history was selected as a method to examine cancer throughout the region. Oral histories provide a detailed account of a topic via detailed, conversational questioning. They tell a narrative using the voices of interviewees, creating individual stories related to a particular subject.^17^,^18^ Oral histories are especially important for preserving history and documenting personal accounts of individuals, which can be used as a tool for future research.^19^ This and other storytelling methods can also provide nuanced, personal insight as well as cultural and historical context to public health issues—perspectives not often achieved through traditional interviews.^20^,^21^ For all of these reasons, they present as a valuable tool in cancer research and offer the opportunity to understand cancer’s impact in Appalachia through personal perspectives and experiences.^22^,^23^

Using an oral history approach, participants were interviewed about problems being faced in Appalachia that contribute to high rates of cancer in the area. Interviews were analyzed using qualitative content analysis, and data were condensed into themes, subthemes, and subtopics. Relational content analysis was used to illustrate relationships between the problems being faced in Appalachia and their contributing factors, with potential solutions to those problems. Unlike conceptual content analysis, which focuses on identifying the presence of themes, relational analysis aims to demonstrate relationships found between themes that emerge and provides a deeper look at individual experiences (in this case, of disease and Appalachian living).^25^,^26^ Ultimately, this study suggests that an oral history approach may allow for a rich understanding of cancer’s impact in Appalachia through personal perspectives of those directly impacted by the disease.

## METHODS

### Study Design

This qualitative research pilot study was based on an oral history approach as a method for data collection using a semi-structured interview guide. Oral histories are a type of narrative research method in which collected data can provide insight into personal perspectives and intentions via individuals’ lived experiences. Insight gained from oral histories can include those related to health, cultural practices and beliefs, and history.^20^,^21^,^23^,^27^

This study aimed to determine whether an oral history approach could be used to understand Appalachian Kentuckians’ perspectives on cancer. The criteria used to assess this outcome were based on whether the data obtained aligned with literature on factors that are associated with the high rates of cancer in Appalachian Kentucky, such as tobacco use, obesity, poverty/income, educational attainment, and decreased access to health care.^3^,^4^,^8^–^14^

### Sampling, Recruitment, and Participants

Purposive sampling was used to identify potential participants. This sampling method engages participants who have information related to the research topic.^28^ Participants (n = 5) were recruited from the community or from community-serving settings, and all participants identified as being from and/or having strong connections to Appalachian Kentucky. Participants included individuals working in cancer-related fields, oncology professionals, and those with personal cancer experience (personal diagnosis and/or diagnosis of a family member). The sample was predominately Caucasian and male (n = 4, 80%). All participants had more than a high school education: some college education, a college degree, and/or an advanced degree.

### Data Collection

In early 2020, four oral history interviews were held in person at the Louie B. Nunn Center for Oral History at the University of Kentucky, and one interview was held by phone due to COVID-19 restrictions. Interviews were semi-structured and allowed for open-ended, conversational responses that were detailed and personalized. The interviews averaged 1 hour 20 minutes in length. Interviewees discussed their personal connections to cancer and responded to questions that sought their views on the issues that could contribute to the high rates of cancer in Appalachian Kentucky and potential solutions to those issues.

### Content Analysis

Content analysis was used to analyze the oral history interviews.^24^,^26^,^29^,^30^ In brief, two researchers individually annotated the data and generated initial coding. Afterwards, researchers met to discuss any discrepancies. The dataset was then reviewed by a third researcher and any discrepancies in coding were noted and reconciled through discussion among the researchers. Lastly, the data were categorized into themes, subthemes, and subtopics. With a small sample size, data saturation may not have been reached; however, common themes, subthemes, and subtopics emerged. Findings were supported by providing participant quotes.

### Relational Analysis

After the content analysis was complete, relational analysis^25^,^26^ was used to identify relationships and/or interconnections among three of the identified themes: problems being faced in Appalachia, factors contributing to those problems, and solutions. The reoccurrence of particular words and phrases was carefully considered within each theme, and related data were analyzed according to the content and context of each finding. Through this approach, relationships were highlighted between the problems being faced in Appalachia, contributing factors, and the solutions to those problems.

### Ethics Approval

This pilot study was conducted as part of an approved University of Kentucky Institutional Review Board Protocol. All procedures were in accordance with the ethical standards of the responsible committee on human experimentation (institutional and national) and with the Helsinki Declaration of 1975, as revised in 2000. Participants consented to the interview and signed waivers to make their interview publicly available for historical, educational, and research purposes.

## RESULTS

### Conceptual Content Analysis

Six key themes emerged from the analysis of the oral history interviews: (1) problems being faced in Appalachia; (2) contributing factors; (3) potential solutions; (4) Appalachian disposition; (5) experiences with and thoughts on cancer; and (6) defining success v. the future without changes (intervention). We identified a further 25 subthemes: high rates of cancer, lack of physicians, lack of access to health care, lack of healthcare literacy, distrust, high rates of smoking, high rates of obesity, economic need, risky behaviors, poor diet, biological and environmental factors, Appalachian fears, lifestyle changes, education and awareness, prevention, increasing access, younger generation voices, community voices, characteristics of people of Appalachia, types of cancer, effects on self/family, generational differences in cancer (a difference in younger views), emotions, what success looks like, and the future without changes. These themes and subthemes are indicated by italics in the summaries below.

#### Theme 1: Problems Being Faced in Appalachia

Participants indicated many problems facing individuals within Appalachian Kentucky (Table 1). Participants described *high rates of cancer incidence* and mortality in the area and discussed how these high rates are, in part, due to the number of neglected cancer cases and cases diagnosed at a late stage. They also noted the *lack of physicians* regionally. Most physicians who decide to practice in Appalachia often do not stay, participants explained. Our participants suggested that the “turnover of physicians in Eastern Kentucky is extremely high.”

Beyond physician recruitment and retention, participants said *lack of access to health care* was a major concern in Appalachia. Individuals in this area have limited local healthcare facilities, and lack of transportation and excessive commute times make seeking care elsewhere difficult. One participant highlighted the difficulty associated with patients traveling from these areas: “When I see a 606 area code, I know they have gone through a lot of trouble to get here.” Individuals in Appalachia live in resource-poor communities and experience delayed cancer treatment due to this lack of access. In a similar vein, *lack of healthcare literacy* was also evident. Participant responses illustrated knowledge gaps between information given by providers (such as a diagnosis) and the understanding of the patient. Participants indicated that it is common for patients to be unfamiliar with their family history and lack pertinent healthcare knowledge overall.

Other common themes related to beliefs and behaviors. *Distrust* for outsiders, such as healthcare providers, is strong within the Appalachian Region. Residents have a lack of trust for those whom they consider “strangers.” In terms of behaviors, study participants described how *smoking* was a social/cultural norm in Appalachia and was common among families. Often, people are influenced or, in some cases, encouraged to smoke. One participant stated, “We were certainly encouraged to smoke at an early age . . . to support the [tobacco] industry.” Aside from high cancer rates, Appalachia also has *high rates of obesity*—what one participant characterized as “excessively high.” Comorbidities such as obesity are associated with an increased risk of cancer.^9^,^10^,^31^

#### Theme 2: Contributing Factors

The participants indicated that many of the problems that exist are associated with issues that the Appalachian area experiences in general (Table 2), including the region’s economic state and lack of available *economic resources*. These can be viewed as contributing factors to high cancer incidence. Participants also reported that Appalachians often partake in *risky behaviors* such as smoking, drug use, self-neglect, and healthcare avoidance without—or despite—knowledge of the consequences. As stated previously, obesity rates are high. Likely connected to this, poor diet was said to be common among individuals from within the region due to lack of access to healthier food options, which often means consuming more processed foods. Other contributing factors that emerged were *biological and environmental factors*. High rates of cancer can be attributed to genetic predisposition or familial mutations and environmental toxins, such as chemicals generated through coal mining.^32^ Appalachians also experience many *fears* that contribute to the deep distrust for those outside the region. Individuals from within the region are conscious of their local accent and regional dialects. They have a fear of being thought of as unintelligent or unsophisticated and worry that they will be misunderstood by those who lack understanding about their ways of life.

#### Theme 3: Potential Solutions

Participants discussed several potential solutions that can address high rates of cancer in the Appalachian Region (Table 3), including *lifestyle changes* to help individuals avoid risky behaviors and cancer *education and awareness* (such as disseminating cancer information through seminars and webinars), increasing awareness of one’s own health, and having an understanding of one’s family health history. Furthermore, enhanced cancer *prevention efforts* arose as a potential solution. Participants discussed the need for learning what prevention is and how to take preventive measures, alongside increasing availability and accessibility of screening. The region also needs *increased healthcare access*. More local healthcare facilities are necessary to bridge the gaps in accessibility. Another way to address cancer is through *younger generations’ voices*. Youth can be used as advocates and thus be a driving force for change. One participant stated, “I’m excited by [cancer training] programs because younger students are really starting to think about cancer as a career. I believe the hope is that they will bring that knowledge back to the community.” Another participant stated that “growing career professionals, oncology professions, from within the region can eliminate provider distrust.” Participants also suggested that Appalachian residents are more receptive to information coming from trusted *community voices* and suggested partnering with community groups and/or local organizations, such as churches, to distribute cancer education/awareness information.

#### Theme 4: Appalachian Disposition

Appalachian people have unique characteristics and ways of life, some of which have negative consequences despite having the potential to be beneficial (see Supplemental Table 1 in “Additional Files”). Participants described five such characteristics, including selflessness, which can contribute to an individual’s self-neglect. Appalachian residents also tend to prioritize family and more often postpone dealing with their personal health problems, feeling as though their health can wait while they care for their family’s needs. Participants described Appalachian people as hard-working individuals who were very appreciative, thankful, and kind-spirited. One participant described Appalachian locals as “fiercely loyal people, very family connected, not wanting to leave when they grow up.” Further, individuals of the region often have a sense of hopelessness around cancer; they commonly approach the topic with a fatalistic mindset. Due to feeling as if their fate has already been decided, they often avoid seeking care.

#### Theme 5: Experiences with and Thoughts on Cancer

Appalachian Kentuckians have unique experiences with and thoughts on cancer (see Supplemental Table 2 in “Additional Files”). The types of cancers most commonly mentioned by study participants included lung, breast, gynecological, prostate, colon, and others (skin, pituitary, oral, brain). Cancer was said to not only affect the quality of one’s life, but also their financial well-being. As one participant stated, “[Cancer] doesn’t just affect the person. It affects the entire family.” Participants also suggested generational differences in how cancer is viewed, saying that younger generations use technology more often, have increased awareness, and are more conscious of their actions related to health and well-being. In terms of emotions, participants reported that cancer evoked feelings of sadness, concern, and that a cancer diagnosis was life changing.

#### Theme 6: Defining Success v. the Future without Changes

Participants defined *success* as decreased rates of cancer by addressing the factors that are driving the cancer disparities (Table 4). For example, increasing access to healthcare facilities, establishing more local facilities, providing services that are within one’s financial capabilities, and having a more educated population overall. Success meant communities that were thriving. In contrast, *without intervention*, participants described a future that would continue along the path of high cancer rates with devastating outcomes due to resource scarcity.

### Relational Content Analysis

**Figure 1:**
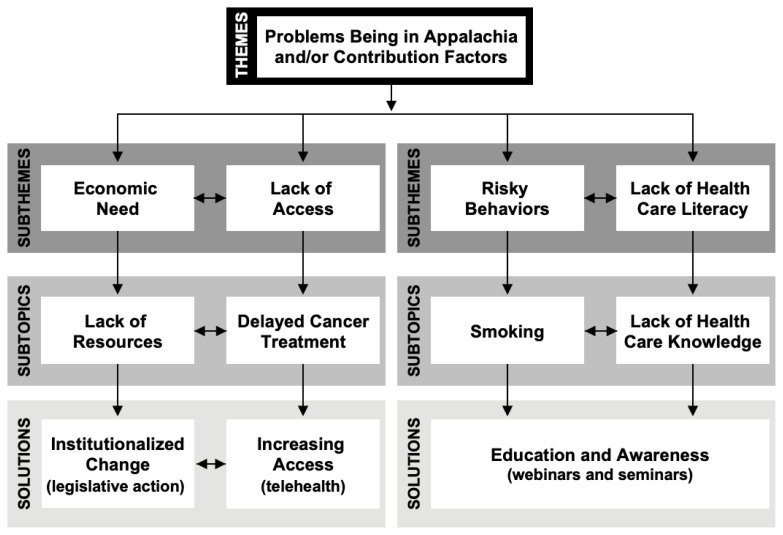
Relational analysis of problems being faced in Appalachia, contributing factors, and their potential solutions

Relational content analysis was used to demonstrate connections between the problems being faced in Appalachia, their contributing factors, and the solutions to these issues proposed by participants (Fig. 1).

One set of relationships was seen in the domain of economics: the region experiences economic disparities with high poverty levels that lead to resource scarcity; this in turn impacts healthcare access, including having few local healthcare facilities. Lack of facilities, coupled with transportation barriers that impede travel, can result in delayed cancer diagnosis and treatment. These issues can be bidirectional: one reason more health care facilities are not available is because of limited financial investments in the area. The lack of such investments perpetuates individual and regional economic hardships, which may limit individual access to or engagement with the healthcare enterprise. The pilot study’s participants envisioned that government intervention could provide funding to the area to, for example, increase access through the expansion of telemedicine.

Risky behaviors and lack of healthcare literacy are also highly interconnected. Residents often do not understand how poor health behaviors can increase one’s risk for developing cancer. Working to increase education levels, including healthcare literacy, could have significant impacts on cancer rates in the area, especially long-term. Overall, many of the causes and consequences of Appalachian Kentucky’s high rates of cancer, along with many of the solutions, are interconnected.

## DISCUSSION

Kentucky experiences the highest overall cancer incidence and mortality rates in the U.S., with the greatest burden of the disease occurring in the Appalachian portion of the state.^3^,^4^ The oral history approach used for this pilot study provides a rich set of data regarding Appalachian Kentuckians’ perspectives on cancer. In fact, the themes, subthemes, and subtopics identified in this pilot study align with known social determinants of health and health behaviors that influence high rates of cancer in the region.^4^,^8^–^14^ This provides preliminary evidence for oral history as a feasible, content-rich approach to study how cancer impacts individuals, families, and communities by revealing in-depth accounts of personal experiences.

Using content analysis of oral history interviews, issues such as lack of physicians, lack of access to health care, lack of health care literacy, and distrust were identified as common problems giving rise to regionally high cancer rates. To address these challenges, participants suggested the following: increase access by recruiting and retaining physicians to practice in the area through incentive programs, increase access to health care by expanding transportation services, and improve healthcare literacy through education and awareness. These suggestions align with known interventions that improve health outcomes. For example, increasing healthcare access can lower the rate of late-stage cancer diagnoses.^33^ Cancer education interventions can also increase cancer literacy, as shown among middle and high school students in Appalachian Kentucky.^34^,^35^ Further, webinars were shown to improve knowledge surrounding cancer disparities in Appalachia and serve as an effective tool for disseminating cancerrelated information to residents in the area.^36^ These methods could be expanded to aid in reducing the cancer burden in Appalachian Kentucky in a culturally appropriate way.

To make a significant and long-term impact on cancer rates in Appalachian Kentucky, interventions suggested by the participants will require intentional and direct action from both external and internal stakeholders, such as state and federal government support, and grassroots efforts by regional and local community members. Efforts are needed to provide funding towards the development of healthcare infrastructure and transportation services (or fuel cards to aid at individual level in transportation to appointments). Additionally, increased cancer literacy can be achieved by implementing policies that would establish programs focused on increasing cancer awareness through cancer education (e.g., webinars, seminars, and events) to residents, community members, and students. In fact, a tailored cancer education intervention has been shown to increase students’ cancer literacy in Appalachian Kentucky.^34^,^35^,^37^ Although implementation of these interventions would require substantial government involvement, these interventions could greatly improve the cancer epidemic facing Appalachians.

### Limitations

Piloting qualitative studies is an important step in the research process. Pilots can help identify potential methodological or data collection issues, determine study effectiveness, and inform the framework of future larger-scale studies.^38^ While the pilot presented here has several strengths, it should be interpreted within the context of its limitations, including its sampling method, sample size, and the demographic makeup of participants. These factors limit the study’s generalizability, and participants’ views and experiences may not be representative of those of all Appalachian people in Kentucky. Due to COVID-19, additional interviews were not able to be conducted for this pilot, resulting in a smaller sample size than anticipated. Small sample size and narrow demographic characteristics of the participants limits the ability to ensure that no new information would be discovered through additional interviews. While it was likely not possible to achieve data saturation within the context of our limited sample size and limited demographic characteristics, we suspect that additional interviews would likely further support the current findings, given that themes, subthemes, and subtopics emerged in alignment with known health behaviors and SDOH that influence cancer incidence in Appalachian Kentucky, and with interventions that could aid in reducing the cancer burden.^4^,^8^–^14^

Additionally, there is potential for interpretation bias in the analysis of the interviews. Appointment of two researchers to independently collect data/analyze the interviews and of a third researcher to review the combined data amidst discrepancies attempted to mitigate this bias.

Despite these limitations, results demonstrate the feasibility of using an oral history approach to obtain rich information regarding individuals’ perspectives on cancer. In the future, aspects of this method can be adopted and modified to improve analysis of both individual and collective healthcare experiences. Future work from this research team is planned to engage a larger sample size through randomized sampling.

## IMPLICATIONS

This pilot study demonstrates the value in using an oral history approach to explore individuals’ perceptions of cancer. Through it, many areas and means of creating culturally appropriate interventions related to Appalachian Kentucky’s cancer disparities—including increasing cancer education in schools and communities, providing education on Appalachian culture and traditions in the onboarding of healthcare workers, increasing funding towards preventive care, enhancing healthcare access, and utilizing the voices of youth and community members—have been identified. Interventions should target lifestyle choices that put one at risk for cancer, such as smoking or avoiding routine screenings, thereby increasing healthy behaviors and healthcare engagement. With financial investment through government funding and implementation of new policies and programs, many of these issues can be addressed to improve the cancer outlook in Appalachian Kentucky. Ultimately, future work using an oral history approach could further probe questions related to how cancer impacts Appalachian Kentucky and how specific, culturally tailored interventions could be deployed a scale to alleviate the region’s cancer burden.

**SUMMARY BOX**

**What is already known about this topic?**

Kentucky has the highest cancer rate in the nation, with the greatest burden of the disease concentrated in the Appalachian Region of the state. This cancer disparity is driven by inequities in key social determinants of health, such as lack of healthcare access and low educational attainment, as well as high-risk health behaviors.

**What is added by this report?**

This pilot study demonstrates the value in using an oral history approach to explore the cancer burden in Appalachian Kentucky. The approach identified several evidence-based interventions that could address the cancer burden: increasing cancer education in schools and communities; providing education on Appalachian culture and traditions in the onboarding of healthcare workers; increasing funding towards preventive care; enhancing healthcare access, including the expansion of telehealth, providing incentives to recruit and retain physicians; and utilizing the voices of youth and community to increase engagement in cancer prevention, screening, and treatment efforts.

**What are the implications for future research?**

Future work using oral history interviews should investigate related research questions with a randomly selected, larger-size sample. Lessons learned in this pilot are currently driving in-depth, follow-on work to study the link between cancer literacy, health behaviors, and healthcare engagement in Appalachian Kentucky. Ultimately, this nascent approach offers a potentially rich pathway for further understanding and contextualizing perceptions of cancer in an at-risk population.

## Supplementary Information



## Figures and Tables

**Table 1:** Theme 1: Problems being faced in Appalachia

Subtheme	Subtopic
High Rates of Cancer	Incidence and mortality
	Late-stage diagnosis
	Neglected cases
Lack of Physicians	Difficulty attracting physicians to the region
	High physician turnover rate/physician retention
Lack of Access to Healthcare	Delayed cancer treatment
	Excessive commute times
	High poverty levels
	Lack of transportation (no vehicle, gas expense, etc.)
	Late-stage diagnosis
	Limited local facilities
	Resource-poor communities
Lack of Healthcare Literacy	Lack of health knowledge
	Unaware of family history
	Unaware of personal medical problems
	Unfamiliar with diagnosis
Distrust	Lack of trust for “strangers”
	Misunderstood by outsiders
High Rates of Smoking	Common among families; generational smokers
	Encouraged to smoke
	Influenced to smoke by others
	Socially acceptable; social norms
High Rates of Obesity	Associated with increased risk of cancer

**Table 2:** Theme 2: Contributing factors

Subtheme	Subtopic
Economic Need	Lack of economic resources
	Poverty
Risky Behaviors	Drug use
	Health care avoidance
	Self-neglect
	Smoking without or despite knowledge of the consequences
Poor Diet	Limited access to healthy food options
	Overconsumption of processed foods
Biological & Environmental Factors	Genetic predisposition
Appalachian Fears	Accent/dialect conscious
	Fear of being talked down to
	Misunderstood by outsiders

**Table 3:** Theme 3: Potential solutions

Subtheme	Subtopic
Lifestyle Changes	Avoid risky behaviors/make healthier lifestyle choices
	Improve dietary choices
Education & Awareness	Emphasize importance of early detection
	Hold seminars and webinars
	Improve dissemination of information
	Increase communication
	Increase research
	Onboarding preparation for healthcare professionals
	Promote awareness of family history
	Provide lectures on prevention
Prevention	Access to healthier lifestyle options
	Increase preventative care funding
	Provide free/accessible screenings
Increasing Access	Create incentive programs for physicians
	Improve physician retention
	Increase the number of local facilities
	Provide transportation services/gas cards
	Recruit and retain physicians
	Recruit community physicians
	Utilize telemedicine and technology to bridge gaps
Younger Generation Voices	Educate youth
	Promote building of young professionals from within
	Stress importance of listening to youth
	Utilize young voices
Community Voices	Use churches as a platform to distribute information

**Table 4:** Theme 6: Defining success v. the future without changes

Subtheme	Subtopic
What does Success Look Like?	Decreased rates of cancer
	Thriving communities
	Increased education
	Increased access to health care
	Increases facilities
	Affordable services
The Future Without Changes	Further lack of resources
	Devastating outcomes
	Increased need
	Continuation of high cancer cases/deaths
