# Supplementary material for: Piloting an Oral History Approach to Investigate Cancer Perspectives Among Residents of Appalachian Kentucky
Source: J Appalach Health. 2023 Apr 1;5(1):95–113. doi: 10.13023/jah.0501.07 (PMC10629891; doi:10.13023/jah.0501.07)
Supplement: Supplementary file 1 [file 5.1.7_Martin_AdditionalFile.pdf]

Supplemental Table 1. Theme 4: Appalachian Disposition.

**Subtheme**	**Subtopic**
Characteristics of Appalachians	Kind<br>Hard-working<br>Appreciative<br>Selfless<br>Appreciative of home/family

Supplemental Table 2. Theme 5: Experiences with and Thoughts on Cancer.

**Subtheme**	**Subtopic**
Types of Cancer	Lung<br>Breast<br>Gynecological<br>Prostate<br>Colon<br>Other (skin, pituitary, oral, brain)
Effects on Self/Family	Decreased quality of life<br>Life-long side effects<br>Financial toxicity<br>Economic well-being of business/state<br>Degradation of family
Generational Differences in Cancer<br>(A Difference in Younger Views)	Changes in cancer care<br>More information now than in the past<br>Increased awareness of routine exams<br>Increased use of technology<br>More health conscious<br>More aware of diet
Emotions	Incredibly sad<br>Concern or lack thereof<br>Life-changing<br>Fatalism
